# Sub-lethal exposure to chlorfenapyr reduces the probability of developing *Plasmodium falciparum* parasites in surviving Anopheles mosquitoes

**DOI:** 10.1186/s13071-023-05963-2

**Published:** 2023-10-03

**Authors:** Prisca A. Kweyamba, Lorenz M. Hofer, Ummi A. Kibondo, Rehema Y. Mwanga, Rajabu M. Sayi, Fatuma Matwewe, James W. Austin, Susanne Stutz, Sarah J. Moore, Pie Müller, Mgeni M. Tambwe

**Affiliations:** 1 Vector Control Product Testing Unit (VCPTU), Environmental Health and Ecological Sciences, Ifakara Health Institute, P.O. Box 74, Bagamoyo, Tanzania; 2 Swiss Tropical and Public Health Institute, Kreuzstrasse 2, 4123 Allschwil, Switzerland; 3 University of Basel, Petersplatz 1, 4001 Basel, Switzerland; 4 Professional & Specialty Solutions, BASF Corporation, Global Development, Public Health Insecticides, Research Triangle Park, NC 27709 USA; 5 Professional & Specialty Solutions, BASF SE, Public Health, 67117 Limburgerhof, Germany; 6 The Nelson Mandela African Institution of Science and Technology (NM-AIST), Tengeru, P.O. Box 447, Arusha, Tanzania

**Keywords:** Chlorfenapyr, Oocysts, Sporozoites, Prevalence, Intensity, Malaria transmission

## Abstract

**Background:**

Pyrethroid resistance in the key malaria vectors threatens the success of pyrethroid-treated nets. To overcome pyrethroid resistance, Interceptor^®^ G2 (IG2), a ‘first-in-class’ dual insecticidal net that combines alpha-cypermethrin with chlorfenapyr, was developed. Chlorfenapyr is a pro-insecticide, requiring bio-activation by oxidative metabolism within the insect’s mitochondria, constituting a mode of action preventing cross-resistance to pyrethroids. Recent epidemiological trials conducted in Benin and Tanzania confirm IG2’s public health value in areas with pyrethroid-resistant *Anopheles* mosquitoes. As chlorfenapyr might also interfere with the metabolic mechanism of the *Plasmodium* parasite, we hypothesised that chlorfenapyr may provide additional transmission-reducing effects even if a mosquito survives a sub-lethal dose.

**Methods:**

We tested the effect of chlorfenapyr netting to reduce *Plasmodium falciparum* transmission using a modified WHO tunnel test with a dose yielding sub-lethal effects. Pyrethroid-resistant *Anopheles gambiae* s.s. with L1014F and L1014S knockdown resistance alleles and expression levels of pyrethroid metabolisers CYP6P3, CYP6M2, CYP4G16 and CYP6P1 confirmed by quantitative reverse transcription polymerase chain reaction (RT-qPCR) prior to conducting experiments were exposed to untreated netting and netting treated with 200 mg/m^3^ chlorfenapyr for 8 h overnight and then fed on gametocytemic blood meals from naturally infected individuals. Prevalence and intensity of oocysts and sporozoites were determined on day 8 and day 16 after feeding.

**Results:**

Both prevalence and intensity of *P. falciparum* infection in the surviving mosquitoes were substantially reduced in the chlorfenapyr-exposed mosquitoes compared to untreated nets. The odds ratios in the prevalence of oocysts and sporozoites were 0.33 (95% confidence interval; 95% CI 0.23–0.46) and 0.43 (95% CI 0.25–0.73), respectively, while only the incidence rate ratio for oocysts was 0.30 (95% CI 0.22–0.41).

**Conclusion:**

We demonstrated that sub-lethal exposure of pyrethroid-resistant mosquitoes to chlorfenapyr substantially reduces the proportion of infected mosquitoes and the intensity of the *P. falciparum* infection. This will likely also contribute to the reduction of malaria in communities beyond the direct killing of mosquitoes.

**Graphical Abstract:**

**Figure:**
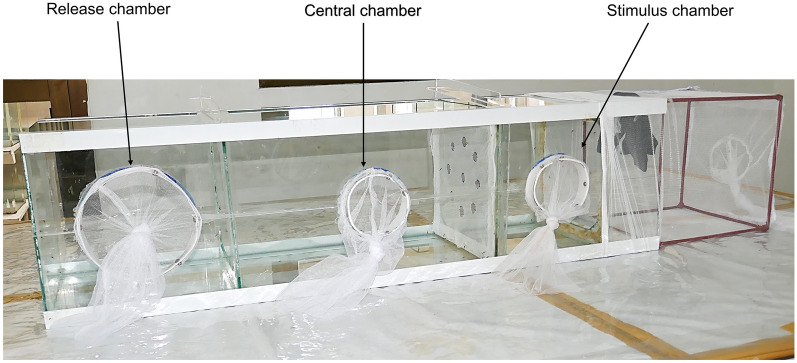

**Supplementary Information:**

The online version contains supplementary material available at 10.1186/s13071-023-05963-2.

## Background

Combining active ingredients (AIs) with two different modes of action on insecticide-treated nets (ITNs) or by using both ITNs and indoor residual spray (IRS) together with different active ingredients is beneficial for the control of insecticide-resistant mosquitoes in endemic settings [1]. One new tool that has recently been recommended for public health is Interceptor^®^ G2 (IG2; BASF SE, Ludwigshafen, Germany), which includes a pyrethroid and chlorfenapyr, a pro-insecticide that is designed to enhance insecticide resistance management [2]. Recent trials of IG2 have demonstrated significant reduction in malaria prevalence compared to pyrethroid only ITNs of 55% in Tanzania [3] and 46% in Benin [4]. Also multiple operational trials have demonstrated the improved control of malaria using IG2 ITNs compared to pyrethroid only ITNs [5]. Pyrethroid-chlorfenapyr ITNs have recently been recommended by WHO for use in the control of malaria areas of malaria transmission where mosquitoes are resistant to pyrethroids [1].

Chlorfenapyr is an Insecticide Resistance Action Committee (IRAC) Group 13 insecticide: a pyrrole chemistry. It uncouples oxidative phosphorylation via disruption of the proton gradient as a protonophore to short-circuit mitochondrial respiration through inner mitochondrial membranes of insect cells so that adenosine triphosphate (ATP) cannot be synthesised and subsequently robs insects of energy resulting in death [2, 6]. While parent chlorfenapyr (CL303630) can be relatively low in toxicity to mosquitoes for some testing modalities, chlorfenapyr requires metabolisation to its pro-insecticidal metabolite, tralopyril (CL303268), in the mosquitoes to elicit lethal effects [6]. This process is dependent on mosquito metabolism, a process that may take time to begin, but once conversion is started the insect’s respiration is increased, which then enhances additional conversion of the parent chlorfenapyr to tralopyril [6]. In nature, mosquitoes will encounter chlorfenapyr while metabolically active, foraging at night when chlorfenapyr is applied to ITNs or after being physiologically or metabolically active from foraging, or while resting on treated walls for blood digestion if clorfenapyr is applied as an IRS. It is also likely that further conversion to the metabolite will occur while gravid mosquitoes are again metabolically active while seeking a place to oviposit.

Metabolic resistance is one of the main mechanisms of pyrethroid-resistance observed in malaria vectors [7]. As pyrethroids are used almost ubiquitously for malaria control, metabolic pyrethroid resistance is widespread throughout sub-Saharan Africa and to a lesser degree to other malaria-endemic areas [8]. Metabolic resistance is where the production enzymes of one or several detoxification gene families, such as cytochrome P450-dependent monooxygenases (P450s), carboxyl-cholinesterases (COEs) and/or glutathione S-transferases (GSTs), are increased and used by mosquitoes to sequester, break down or export insecticides so they can survive exposure to lethal concentrations [9]. While this metabolism is a detoxification process, it can increase the potency of a pro-insecticide that is metabolised from a parent molecule and may, therefore, be exploited as a means to control metabolically resistant insect populations [2].

It is known that exposure to doses of insecticides that are sub-lethal for insecticide-resistant mosquitoes can interfere with parasite development inside mosquitoes [10], while the underlying mechanisms remain unknown. Owing to the mode of action of chlorfenapyr on mitochondrial respiration in many organisms [6], we hypothesised that chlorfenapyr may also interfere with the metabolic mechanism of the *Plasmodium* parasite in infected *Anopheles* mosquitoes and thereby interfere with malaria parasite development in mosquitoes that were exposed to sub-lethal doses of chlorfenapyr.

For this reason, we developed and evaluated a modified WHO tunnel test to expose mosquitoes to a sub-lethal dose to measure whether pre-exposure to chlorfenapyr impacts the prevalence and intensity of infection of *Plasmodium falciparum* among *Anopheles gambiae* s.s. mosquitoes that survived exposure to chlorfenapyr-treated nets.

## Methods

### Study design

The study was a single (statistician) blinded full factoral design using a modified WHO tunnel test to (i) determine the dose of chlorfenapyr on an ITN that was sufficiently low for at least 50% of *An. gambiae* s.s. mosquitoes to survive for up to 9 days, corresponding to the extrinsic incubation period for oocysts, after exposure, and (ii) measure whether pre-exposure to the chlorfenapyr ITN impacted the prevalence or intensity of oocysts and sporozoites in *An. gambiae* s.s. mosquitoes fed on blood containing *P. falciparum* gametocytes.

### Mosquito rearing

*Anopheles gambiae* s.s. from the Kisumu KDR strain were obtained from the Centers for Disease Control in Kisumu, Kenya, in 2018 and maintained in colony at the Ifakara Health Institute (IHI), Bagamoyo. The strain has L1014F and L1014S knockdown resistance alleles with expression levels of pyrethroid metabolisers CYP6P3, CYP6M2, CYP4G16 and CYP6P1 confirmed by RT-qPCR [11, 12]. This strain was used because previous experiments had demonstrated the strain to have long survival and good development of parasites (Hofer unpublished). The Kisumu KDR mosquito larvae were maintained at a density of 200 per litre of water and fed 0.3 g per larva on Tetramin fish food (Tetra Ltd., UK). For colony maintenance, the adult mosquitoes were fed on cow blood between 3 and 6 days after emergence for egg development using a Hemotek^®^ membrane feeder (SP-6 System, Hemotek Ltd., Blackburn, UK). Mosquitoes were provided with autoclaved 10% sucrose solution ad libitum. Temperature and relative humidity within the insectary are maintained between 27 ± 2 ℃ and 60–85% following the MR4 guidance [13]. Strict hygiene measures were maintained to ensure the colony was free of microsporidia.

### Experimental nets

Untreated 100 denier multi-filament polyester nets and treated multi-filament polyester nets coated with 100 mg/m^2^ and 200 mg/m^2^ chlorfenapyr were used in the study. Nets were provided by BASF SE (Ludwigshafen, Germany).

### Modified WHO tunnel test

**Fig. 1:**
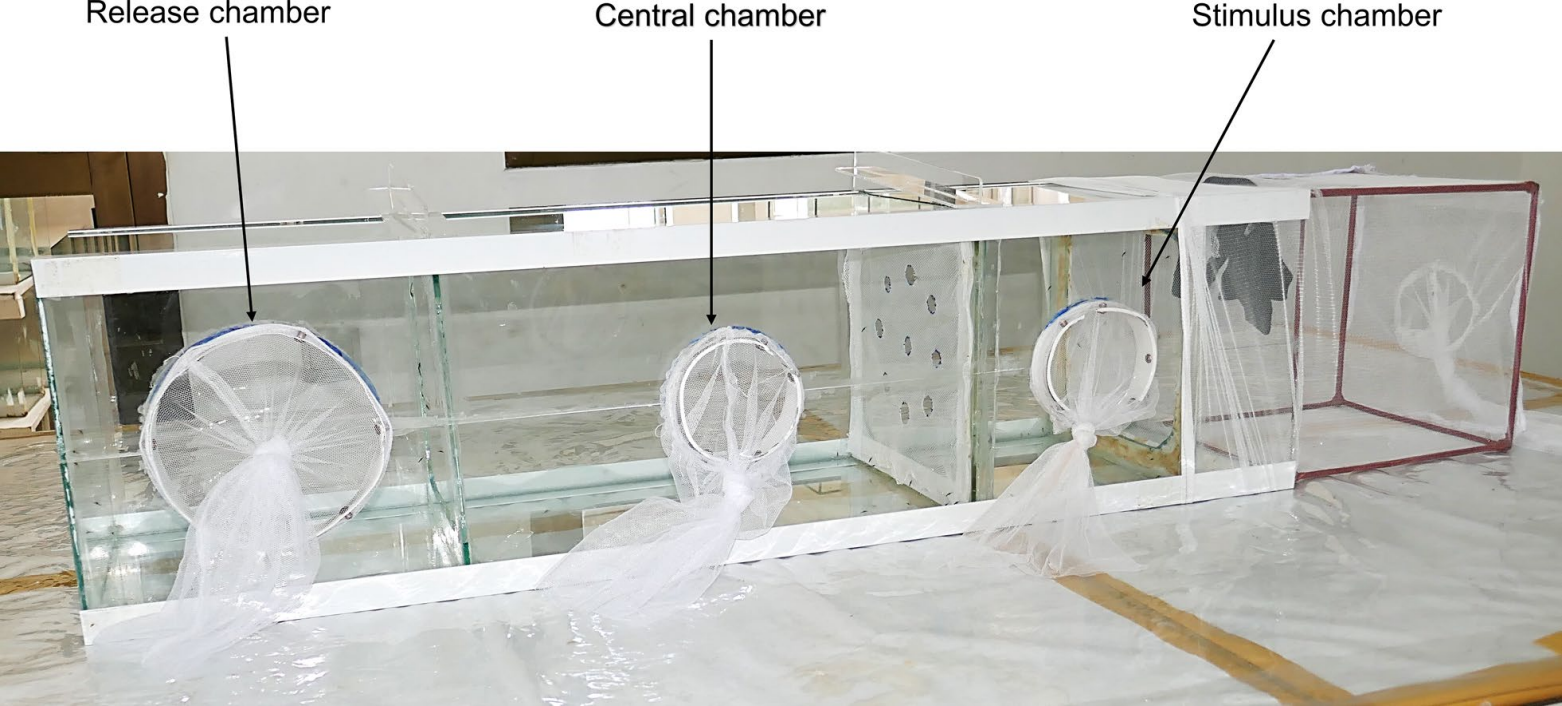
Modified WHO tunnel assay. The main sections are the release, central and stimulus chambers. The partition between the central chamber and the response chamber was designed to attach individual test-treated nets or control. To protect mosquitoes from escaping, the extension cage was made from a Safi net with 100 deniers that do not allow the mosquito to pass and socks placed in the stimulus chamber as a bait to lure mosquitoes towards the test-treated nets or control

The modified tunnel test set-up was first used in testing the behaviour of mosquitoes around vector control tools by Thiévent et al. [14] and was further modified to fit the purpose of the present study. Here, the tunnel measured 25 cm × 25 cm × 122 cm and was made of glass. The tunnel was divided into three sections, including a release chamber, a central chamber and a stimulus chamber (Fig. 1). The specific modifications were (i) the use of worn socks as a stimulus; (ii) adding an extension cage (25 cm × 25 cm × 25 cm) with the worn sock inside; (iii) removing the partition between the release and central chamber to increase the number of mosquitoes accessing the stimulus chamber.

### Experimental procedures

In preliminary tests, mosquito survival was assessed over 9 days after exposure to the untreated and chlorfenapyr-treated net samples. Nine days were chosen because sufficient numbers of chlorfenapyr exposed mosquitoes have to survive long enough for malaria parasites to develop into oocycts, a process that takes around 8 days after feeding. Six replicates were conducted for each dose of chlorfenapyr and a negative control to monitor assay quality. In each replicate, 100 mosquitoes were released from paper cups into the release chamber at 22:00 h. The following morning at 06:00 h, mosquitoes from all chambers were collected and aspirated into paper cups in batches of 50 using a mouth aspirator (J.W. Hock & Co., Gainesville, FL, USA).

Following the exposure in the tunnel test, mosquitoes were transferred to the feeding room and covered with a black cloth for 1 h before receiving a non-infectious blood meal donated by one of the authors (PAK). The non-infectious blood was administered to mosquitoes through water-jacketed glass feeders (14 mm Ø, Chemglass, Vineland, NJ, USA) covered with Parafilm^®^, connected to a circulating water bath (39 ℃, ELMI, Newbury Park, CA, USA) via plastic tubing for 15 min.

After feeding, the cups were transferred to 30 cm × 30 cm × 30-cm Bugdorm plastic cages (Megaview Science Co., Ltd, Taiwan) and placed in a climatic chamber (S600PLH, AraLab, Lisbon, Portugal). Mosquitoes were left for 48 h without sugar for unfed mosquitoes to die. Thereafter, mosquitoes were supplied daily with autoclaved 10% sucrose solution on a cotton pad and kept at 75 ± 2% relative humidity and 27 ± 1 ℃ at a 12:12 h dark:light cycle. Mortality was recorded daily for a over 9 days.

Once the optimal experimental netting was identified, the assays were repeated using blood drawn from gametocytemic carriers. Five mililitres of infectious blood was collected from microscopically confirmed gametocytaemic carriers with gametocyte densities greater than three gametocytes per 500 red blood cells [15]. Autologous serum was removed from the whole blood and replaced with pre-warmed malaria-naïve AB serum. Before feeding and after exposure in the tunnel test, mosquitoes were kept in paper cups with access to deionised water for 1 h. Then, mosquitoes were allowed to feed for 15 min on water-jacketed glass feeders as above. On average, 600 mosquitoes received a blood meal from each participant. Dead mosquitoes were removed from the cup by aspiration 48 h post blood meal. The mosquitoes were provided with cotton soaked with 10% sucrose solution that was changed daily. Eight days post infection (dpi), one third of the mosquitoes were dissected and midguts were stained using 1% mercurochrome solution before examination for presence of oocysts. The remaining mosquitoes were kept up to 16 dpi and were extracted for molecular diagnosis and quantification of *P. falciparum* infection mosquito stages from the mosquito heads and thoraces (sporozoite stage) using RT-qPCR [16]. While the oocysts were actually counted microscopically, numbers of sporozoites were estimated using a plasmid containing a fragment of the 18S rDNA gene (GenBank: AF145334) from *P. falciparum* (BEI Resources, NIAID, MRA-177). Plasmid copy numbers per µl were calculated as described elsewhere [17] and plasmid standard curves were prepared using serial dilutions over eight magnitudes assuming an average of six copies of the 18 s rDNA gene sequence per parasite genome [18]. Each concentration from the serial dilution (standards) was run in triplicate to determine qPCR efficiency, limit of detection, slope and y-intercept for absolute quantification of *Plasmodium* DNA in sporozoite-infected mosquito samples.

### Study participants

The study was conducted in Bagamoyo district located in the coastal region of Tanzania between September 2021 and August 2022. The target populations were males and females aged 6–40 years from the villages of Wami-Mkoko and Ludiga. Participants who met the inclusion criteria (i.e. asymptomatic, consented 6–40 year olds with microscopically detectable gametocytes) were recruited for blood-drawing at the IHI malaria transmission facilities in Bagamoyo. Gametocytes were counted against 500 white blood cells in thick smears. Four of eight individuals tested positive for gametocytes and had more than three gametocytes per 500 white blood cells. Only participants with gametocyte densities of 128 gametocytes per/µl were enrolled for blood drawings. The density of gametocytes was calculated from an estimated leukocyte density of 8000 per µl of blood.

### Statistical analysis

Data cleaning and analysis were done in STATA 17 software (StataCorp LLC, College Station TX, USA). Descriptive statistics were used for data summaries, whereby mean mortality and proportion of oocyst and sporozoite-infected mosquitoes of those alive at day 8 and 16, respectively, with 95% confidence intervals are presented. For parasite intensity (oocysts or sporozoites), median with minimum and maximum values are presented.

To assess the effect of the low- and high-dose chlorfenapyr-treated netting on mosquito mortality, mixed-effects logistic regression with a binomial error distribution and a log link function was used. Replicate and dose were included as fixed effects in the model and experimental day was fitted as a random effect.

To assess the effect of sub-lethal chlorfenapyr exposure on *P. falciparum*, mixed-effects logistic regression was used with treatment as a fixed effect and study participants included as a random effect. Prevalence of infected mosquitoes at oocyst and sporozoite stages was evaluated using a generalised linear mixed-effects model with a binomial error distribution and a log link function. For oocyst and sporozoite intensities, models with a negative binomial error distribution and a log link function were fitted.

## Results

### Selection of chlorfenapyr dose

While the mortality rates were significantly higher in the chlorfenapyr-exposed mosquitoes, the mortality rates between the 100 and 200 mg/m^2^ chlorfenapyr net were similar and < 50% (Table 1). Therefore, the following experiments with infected blood were conducted using the 200 mg/m^2^ chlorfenapyr net samples.

**Table 1:** Mortality rates in blood-fed pyrethroid-resistant *Anopheles gambiae* s.s.

Chlorfenapyr dose [mg/m^2^]	*n*^*2*^	Mortality rate [%] (95% CI)	Odds ratio^1^ (95% CI)	*P*-value
0	600	29 (22–35)	1	
100	600	36 (24–49)	1.46 (1.12–1.90)	0.005
200	600	43 (35–51)	1.87 (1.44–2.43)	< 0.001

Upon exposure to chlorfenapyr-treated netting

^1^Odds ratios between control and chlorfenapyr-treated nets. Mortality rates and odds ratios together with the 95% confidence intervals (95% CIs) were estimated using generalised linear mixed-effects models with a binomial error distribution and log link function

^2^Number of mosquitoes tested

### Effect of chlorfenapyr on infection rates

**Fig. 2:**
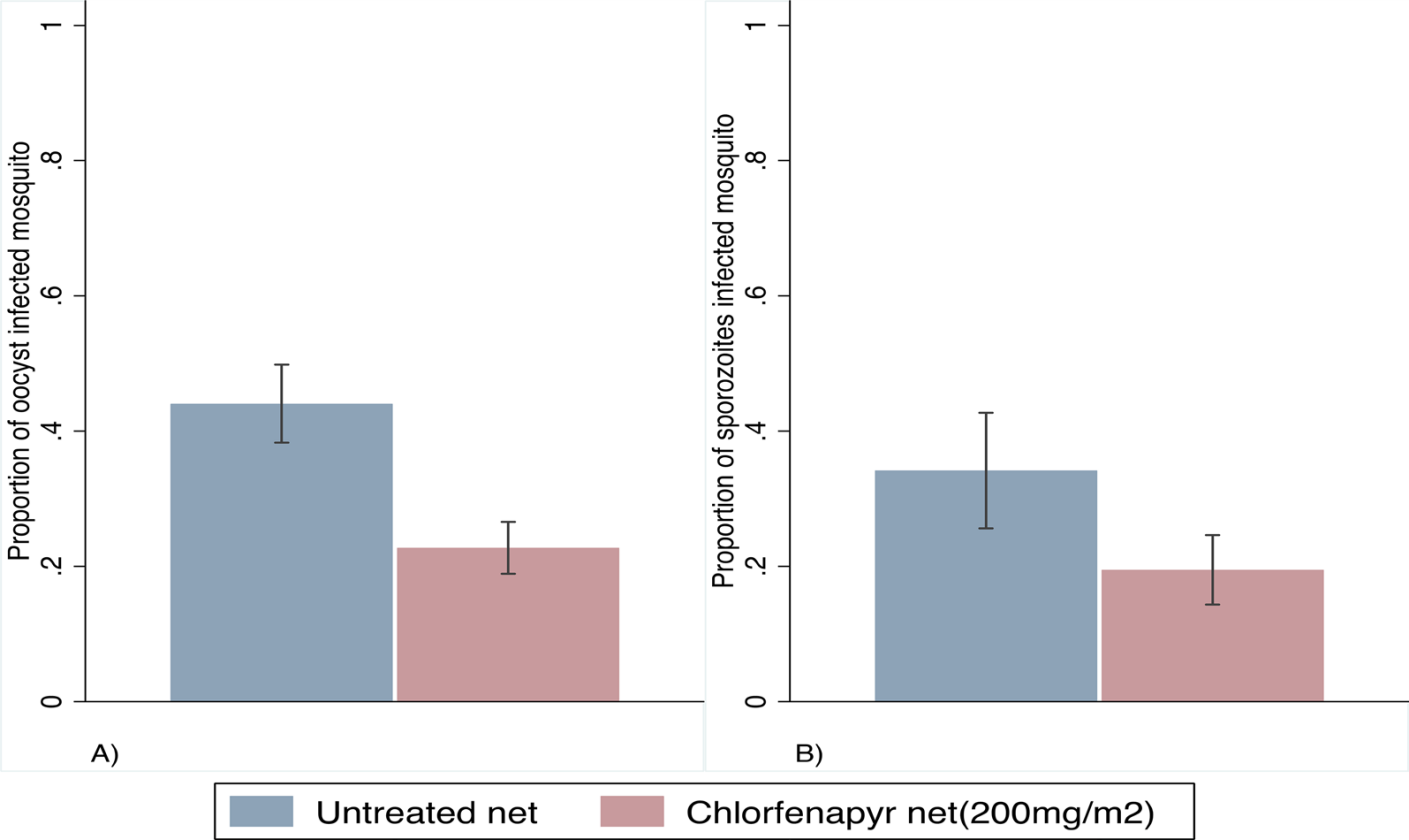
Effects of chlorfenapyr on *Plasmodium falciparum* infection rates in pyrethroid-resistant *Anopheles gambiae* s.s. **A** Oocyst infection rates determined microscopically. **B** Sporozoite infection rates measured by RT-qPCR

In total, we dissected 748 mosquitoes (chlorfenapyr net = 462 and untreated net = 286) on day 8 post-infection. We recorded 105 and 126 oocyst-infected mosquitoes in the chlorfenapyr arm and untreated arm, respectively. The remaining 351 (chlorfenapyr = 231 and untreated net = 120) mosquitoes were extracted for sporozoite detection on day 16 post-infection of which 45 and 41 mosquitoes were infected with sporozoites in the chlorfenapyr arm and untreated arm, respectively. The chlorfenapyr-exposed mosquitoes had much lower oocyst and sporozoite infection rates than those exposed to the untreated net (Fig. 2A, B). The odds of oocyst infection were 0.33 (95% CI 0.23–0.46) lower in the chlorfenapyr net compared to untreated net while the odds for sporozoite infection were 0.43 (95% CI 0.25–0.73).

### Effect of chlorfenapyr on oocyst and sporozoite intensity

**Fig. 3:**
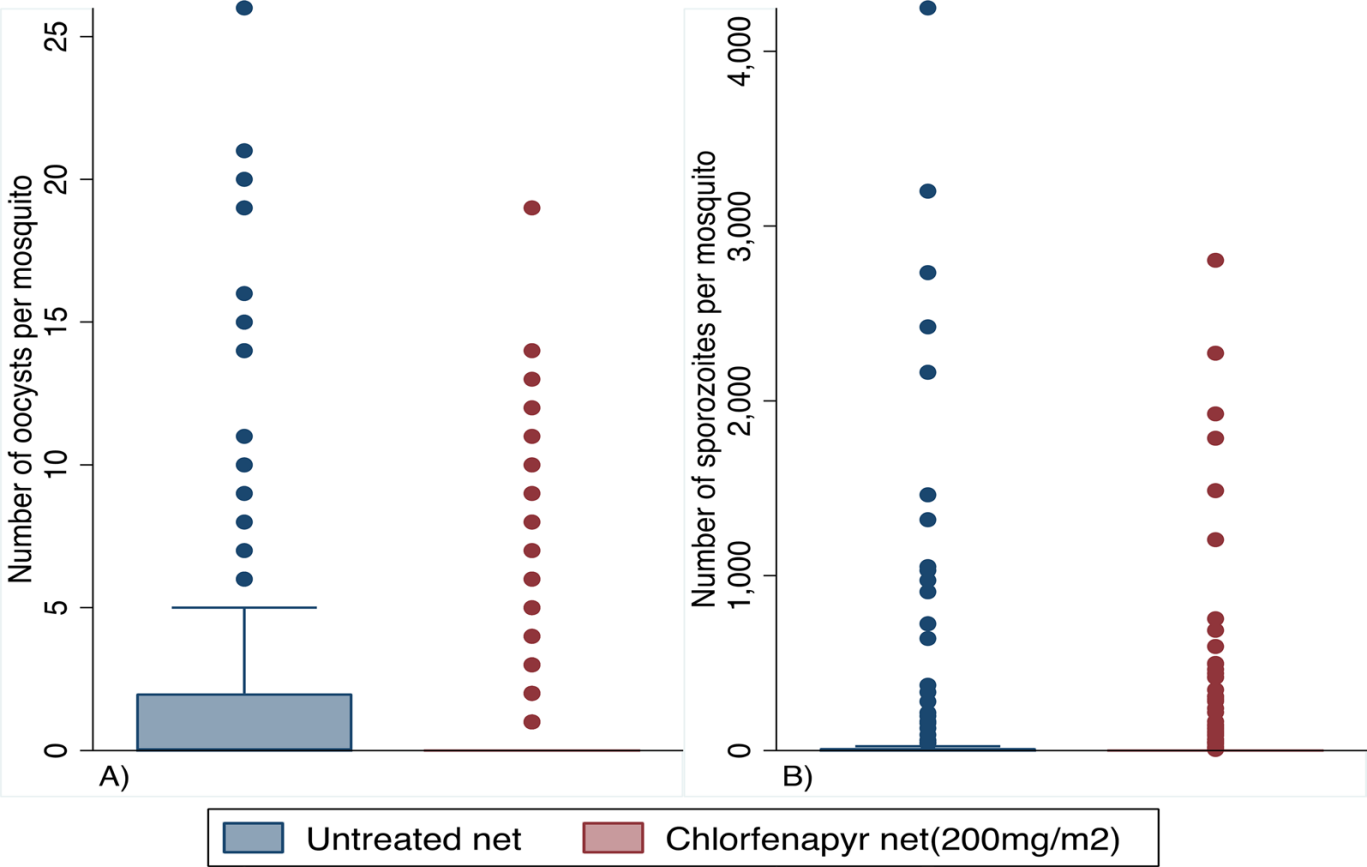
Effects of chlorfenapyr on *Plasmodium falciparum* infection intensities in pyrethroid-resistant *Anopheles gambiae* s.s. **A** Oocyst infection intensity determined microscopically.** B** Sporozoite infection intensity measured by RT-qPCR

In addition to the decreased oocyst infection rate in the chlorfenapyr exposed mosquitoes, the oocyst intensity was also much lower (Fig. 3A, B). Here, the incidence rate ratio (IRR) was 0.30 (95% CI 0.22–0.41) and statistically significantly different from 1 (GLMM, *P*-value < 0.003). In contrast, the IRR in the sporozoite rate was statistically not significantly different from 1 [0.41 (95% CI 0.13–1.30)].

## Discussion

These results describe for the first time an effect of a chlorfenapyr-treated net on the development of wild malaria parasites in pyrethroid-resistant *Anopheles* mosquitoes. In this study we have used a modified WHO tunnel assay to pre-expose mosquitoes to a chlorfenapyr-treated net and have them to actively fly around, thus allowing to study the impact of chlorfenapyr on malaria parasites at sub-lethal exposure. When the exposed mosquitoes were subsequently offered a blood meal containing malaria gametocytes from blood donors in Tanzania, half of the mosquitoes did not develop oocysts or sporozoites despite the fact that the mosquitoes survived the extrinsic incubation period.

Exposure of mosquitoes to the net treated with 100 mg/m^2^ or 200 mg/m^2^ chlorfenapyr induced similar mortality rates. Therefore, experiments were conducted using 200 mg/m^2^ chlorfenapyr dose. It was advantageous to evaluate 200 mg/m^2^ of chlorfenapyr because this reflects the amount present in the IG2 mosquito net that has alpha-cypermethrin (100 mg/m^2^) and chlorfenapyr (200 mg/m^2^) coated onto polyester netting. The results from this experiment likely reflected concentrations that would be encountered under field conditions. However, neither nets with pyrethroid nor a combination between pyrethroid and chlorfenapyr was included as these have also been shown to reduce the prevalence and intensity of infection among mosquitoes exposed to sublethal concentrations [10].

Bioassay design is an important factor when evaluating chlorfenapyr; therefore, tests with ‘free-flying’ mosquitoes conducted at night are more appropriate for evaluation of chlorfenapyr products as they predict the results of gold standard experimental hut trials better. Indeed, hut trials generally demonstrate high efficacy against metabolically resistant mosquitoes in both East [19–23] and West Africa [24–29] as well as against *Aedes aegypti* in Mexico [30], while cone bioassays or CDC bottle assays, for example, where mosquitoes remain inactive show poor performance of chlorfenapyr [23, 31–33]. Use of a resistant strain is also clearly important since we have observed little difference in mortality between pyrethroid only ITNs and IG2 ITNs [34] when susceptible strains were used, as has also been seen by several other authors [19, 28]. The enzymatic transformation of chlorfenapyr to tralopyril is dependent on mosquito metabolism [6] and, in nature, mosquitoes will encounter chlorfenapyr while actively host-seeking at night. The modified WHO tunnel test, therefore, allows mosquitoes to fly around at night when their metabolic enzymes are upregulated [35], ensuring greater conversion of chlorfenapyr to tralopyril. Additionally, efforts to evaluate chlorfenapyr and tralopyril residues after mosquito exposure to chlorfenapyr on a net are underway.

While several studies have shown a reduction in parasite prevalence or intensity due to sublethal exposure of resistant mosquitoes to bendiocarb, DDT [36] or pyrethroids [10, 37], the mechanism for this is not clear. Possible mechanisms could be that the insecticide modifies mosquito physiology such as increasing oxidative stress leading to increased insecticide susceptibility or inhibits the development of the parasite [36, 38, 39]. However, the principal metabolite of chlorfenapyr through n-dealkylation was known to elicit lethal effects on *Plasmodium *in vitro [40]. Similar evidence has been demonstrated where specific anti-malarial drugs such as atovaquone were assessed by *An. gambiae* tarsal contacts to treated surfaces at low doses to demonstrate rapid and complete transmission blocking to *P. falciparum* [41]. The limitations of these earlier studies have been their focus exclusively on laboratory studies and different substrates rather than actual netting used in the manufacture of current or new ITNs. An additional study has shown that direct ingestion of chlorfenapyr in a sugar meal significantly reduces the prevalence and intensity of infection in mosquitoes [42], though exposures to chlorfenapyr on ITNs that afford bioactivation of chlorfenapyr via tarsal contacts to elicit mortality and impairments *Plasmodium* are both logistically favoured and a recommended delivery mechanism as outlined by WHO.

This study showed that chlorfenapyr delivered on an ITN substantially reduces the proportion of *Plasmodium*-infected mosquitoes and the intensity of infection at sub-lethal doses. As insecticide-treated nets age, bio-efficacy is reduced because of insecticide washed from the ITN surface during normal user washing [43]. Therefore, it may be possible that some mosquitoes escape a toxic dose as the insecticidal content of the ITNs wanes after several years under user conditions [44]. The observed effect of chlorfenapyr in reducing parasite prevalence and intensity is particularly useful as there is emerging evidence that insecticide-resistant vectors are more competent to *Plasmodium* [45] because they either are more likely to live longer than their susceptible counterparts that are killed by insecticide [38] or have lower immunity to parasites [46], even though many insecticide resistance mechanisms impose a fitness cost on mosquitoes in the absence of selection pressure [47]. This study using wild *Plasmodium* isolates is the first to demonstrate that vector control products based on chlorfenapyr may have additional malaria control benefits by impacting the parasite and vector simultaneously.

The effect of chlorfenapyr on *P. falciparum* observed in the present study could also explain to some extent why IG2 ITNs showed additional efficacy compared to ITNs that contain a combination of a pyrethroid and PBO or a pyrethroid and pyriproxifen. Although it is conceivable that chlorfenapyr directly affects *P. falciparum* in mosquitoes exposed to the insecticide, the observed phenomenon may also be due to indirect effects, such as increased insecticide susceptibility in infected mosquitoes, or a combination of direct and indirect mechanisms. Therefore, additional studies are required to further explore the nature of this mechanisms and chlorfenapyr’s overall ability to affect malaria transmission.

## Conclusion

We demonstrated that sub-lethal exposure of pyrethroid-resistant mosquitoes to chlorfenapyr substantially reduces the proportion of infected mosquitoes and the intensity of the *P. falciparum* infection. This will likely also contribute to the reduction of malaria in communities beyond the direct killing of mosquitoes.

### Supplementary Information

**Additional file 1.** Chlorfenapyr exposure data.

**Additional file 2.** Chlorfenapyr Oocyst + Sporozoite data.

## Data Availability

The data generated during this study are included in this published article and its Supplementary Information files.
